# Next of kin's perceptions of the meaning of participation in the care of older persons in nursing homes: a phenomenographic study

**DOI:** 10.1111/scs.12636

**Published:** 2019-01-03

**Authors:** Kajsa Ekström, Sanna Spelmans, Gerd Ahlström, Per Nilsen, Åsa Alftberg, Birgitta Wallerstedt, Lina Behm

**Affiliations:** ^1^ Faculty of Medicine Department of Health Sciences Lund University Lund Sweden; ^2^ Department of Medical and Health Sciences Division of Community Medicine Linköping University Linköping Sweden; ^3^ Faculty of Health and Society Department of Social Work Malmö University Malmö Sweden; ^4^ Faculty of Health and Life Sciences School of Health and Caring Sciences Linnaeus University Kalmar Sweden; ^5^ Centre for Collaborative Palliative Care Linnaeus University Växjö Sweden

**Keywords:** 80 years or older, aged, next of kin, nursing home, participation, phenomenography

## Abstract

**Background:**

Being involved in the care of a loved one is a desire of many next of kin. However, according to several studies of the perceptions of nursing home staff, the involvement of next of kin is not an obvious part of care. To be able to involve next of kin in care at nursing homes, the perceptions of what participation means are an important piece of knowledge. The aim of this study was therefore to describe variations in next of kin's perceptions of the meaning of participation in the care of older persons living in nursing homes.

**Methods:**

Eighteen next of kin of older persons living in ten nursing homes in Sweden were recruited for interviews. The study design was based on a phenomenographic approach, focusing on the qualitatively different ways in which a person perceives, experiences or conceptualises a phenomenon or certain aspect of reality.

**Results:**

Five categories emerged from analysis of the interviews, representing the next of kin's perceptions of the meaning of participation in the care of older persons in nursing homes: be present; communicate; monitor; do practical tasks; and to represent. The next of kin expressed meanings that belonged to more than one category, and the categories were interdependent.

**Conclusions:**

Our results indicate that there are several meanings of next of kin's perceptions of participation at nursing homes. Nursing home staff's knowledge of these perceptions is important to enable next of kin to participate according to their own preferences.

## Background

The time spent in nursing homes today is decreasing because older persons live in their own homes as long as possible 1. Nursing homes are a common place to die 2, 3, 4, which implies that older persons living in nursing homes are in their final stage of life and are the most frail persons in our society. Being involved in the care of a loved one is a desire of many next of kin 5 and is considered a cornerstone of palliative care. However, according to nursing home staff 6, the involvement of next of kin is not an obvious part of care.

To move to a nursing home means loss of both independence 7 and privacy 8. For the older person and for the next of kin, the move may imply a double transition of both accepting help 9 and becoming aware of the imminent death of a loved one 10, 11. Next of kin have mixed feelings after older persons have moved to a nursing home, experiencing both a sense of relief and frustration about having to take responsibility when the help is insufficient 11. While many next of kin continue to participate in the care of the older person by doing the same things as they did at home 11, other next of kin find that they need to take greater responsibility at the nursing home than they would want to 11, 12, and some describe a state of dependency of the professionals 5. According to next of kin, participation is described as a way to handle anxiety and maintain control in difficult situations, and not being involved could induce feelings of dissatisfaction and guilt 5. The exclusion of next of kin from care can lead to feelings of alienation, reduced meaningfulness, a less ‘good death’ for the older person and a difficult grieving process for the next of kin 13. Thus, the involvement of next of kin should be an obvious part of care 6. However, the communication between next of kin and the nursing home staff can be complicated 5, 14, 15, 16. For example, next of kin feel that it is their duty to monitor the care at the nursing home, but at the same time, they are afraid to be perceived as supervisors by the staff 16, 17. In addition, next of kin to older persons at nursing homes 16 have reported that they need to be careful to avoid implying that the older person was poorly treated but also that they had an important role giving feedback to the staff 17. Thus, knowledge of what participation in care means for the next of kin is important to improve the collaboration between staff and next of kin.

A few previous studies have focused on the participation of next of kin in nursing homes. They showed that participation is expressed by the next of kin in terms of exchange of information 18, 19, 20, 21, 22, 23, practical help in caring for the old person 18, 19, 20, 21, 22, being present at the nursing home 17, 18, 21, being respected for their knowledge 20, 22, 23, 24, 25, having a good relationship 20, 22, 25, being admitted as part of the team 17, 18, 25 and trusting the staff 18. However, some of these studies were conducted more than 15 years ago, and the characteristics of the residents living in nursing homes have changed considerably since then. Residents in nursing homes today are older and more frail than previously, which may have relevance for how the next of kin want or are able to participate in care. This study addresses this important knowledge gap. Thus, the aim of this study was to describe variations in next of kin's perceptions of the meaning of participation in the care of older persons living in nursing homes.

## Methods

### Design

A qualitative method with a phenomenographic approach was chosen to explore the next of kin's perceptions of the meaning of participation in care at nursing homes. The phenomenographic approach aims to define the qualitatively different ways in which a person perceives, experiences or conceptualises a phenomenon or a certain aspect of reality 26. People's understanding of a phenomenon is to be found in a limited number of qualitatively different ways. From a phenomenographic point of view, the ambition is to reflect these understandings and not judge them as right or wrong 27.

### Study setting

The study was undertaken in Sweden. The welfare system in Sweden is largely funded by taxes and provides access to health care for everyone. The elderly care is based on each person's need of support. Nursing homes are an individual accommodation provided under the Social Services Act 28, where staff are available around the clock and the municipality is responsible for care up to the medical level. Staff in nursing homes consist of assistant nurses, registered nurses, occupational therapists and physiotherapists, and the physicians make regular visits. Moving to a nursing home typically happens when a person is too sick or frail to be able to manage an independent life at home 29.

### Study participants and recruitment procedure

Eighteen next of kin to older persons were recruited from ten nursing homes in southern Sweden in 2016. The nursing homes were situated in both rural and urban areas to balance the socio‐economic diversity of the populations in the geographic areas. One contact person (a nurse aid or the manager at the nursing home) was recruited at each nursing home, and this person was responsible for selecting and asking the next of kin if they wanted to be part of a study. The inclusion criteria were next of kin to an old person living in a nursing home and spoke Swedish as their first language. The eighteen next of kin were strategically sampled to include persons who differed in terms of age, sex and relation to the old person. The contact person gave the next of kin verbal information about the study, and they were given the opportunity to ask questions about the study. If interest to participate in the study, the contact person handed over the next of kin's name and telephone number to the researchers. One researcher contacted the next of kin by telephone and gave verbal information about the study. If consent was given, a time and place for an interview were booked. For characteristics of the included next of kin, see Table 1.

**Table 1:** Characteristics of the study participants (n = 18)

Characteristics	Total n = 18 n %	
Age (range 52–77, median 65)		
52‐ 64	9	50
65–77	9	50
Gender		
Female	13	72
Male	5	28
Relationship to older person		
Husband/wife	5	28
Daughter/son	12	67
Sibling	1	5
Education		
Elementary school	7	39
High school	3	17
University	7	39
Unknown	1	5
Marital status		
Married/living together	13	72
Unmarried/divorced	5	28
Working		
Yes, full time	6	33
Yes, part time	4	22
No	7	39
Unknown	1	6
Frequencies of visit to the older person		
Every day	4	22
Once or more per week	13	72
Unknown	1	6

### Data collection

At the interview, both verbal information and written information were given, and the next of kin signed written consent to participate. The interviews were made as a dialogue that was designed to capture the next of kin's perceptions of the meanings of the phenomena of interest 30. A semi‐structured interview guide was constructed with an initial open‐ended question: ‘What does participation in the care of a next of kin mean to you?’ After the initial question, follow‐up questions were asked. The number and formulation of follow‐up questions depended on the richness of the participant's answer to the open‐ended question. The interviewer adapted to what the interviewees said, and the follow‐up questions were used to obtain as rich information as possible 30. The interviews were conducted by four researchers in the wider research group (registered nurses, all with experience of conducting qualitative interviews in geriatric care; including one with experience from palliative care). Two of the interviewers are authors of this paper (LB and BW). The four researchers had regular meetings throughout the study to discuss the interviews and achieve consistency. The interviews were recorded digitally and lasted for an average of 36 minutes (range: 20–60 minutes). All interviews were transcribed by the first and second authors (KE and SS) before initiating the analysis.

### Data analysis

A phenomenographic analysis was used, in accordance with descriptions by Dahlgren and Fallsberg 31. The analysis was conducted according to the following steps. First, all the interviews were listened to and read carefully three times by the first (KE) and the second author (SS) to obtain an overview (familiarisation). The second step was a selection procedure in which qualitatively meaningful quotes that dealt with the perceptions of participation were extracted from all interviews to achieve a concentrated and representative version of the entire dialogues. In the third step (comparison), the extracted quotes were contrasted to each other to uncover sources of variation or agreement. Similar quotes were grouped together. The next step (articulating) involved describing the essence of the similarity within each group. A labelling step gave the categories names that corresponded to the essence of their meaning. The last step (contrasting) consisted of comparisons of the categories with each other to describe the unique character of each category. Each category had to be qualitatively unique. There was continuous interplay in the entire process between the various steps of the analysis. The analysis was conducted by the first (KE) and the second author (SS) before joint discussions with the last author (LB). The other authors (GA, PN, ÅA and BW) scrutinised the categories, before consensus was reached.

### Ethical considerations

The study is guided by the research ethical principles for medical research 32. Written, informed consent was obtained from all the participants before the start of the study. The next of kin were informed that they had the right to withdraw from the study without any consequences. Data are presented on a collective level to protect the identity of the participants. The Regional Ethics Review Board in Lund, Sweden, approved the study (no 2015/69).

## Results

Five categories emerged from the analysis of the interviews, representing the next of kin's multiple meanings of participation in the care of older persons in nursing homes (Table 2). The five categories were *A: Be present*;* B*:* Communicate*;* C: Monitor*; D: *Do practical tasks;* and *E: Represent*. The meanings are interdependent. Table 2 shows that the next of kin conveyed perceptions that belonged to more than one category, suggesting that some participants had a more complex understanding of the phenomenon than others. The categories are illustrated with quotations from the interviewed next of kin.

**Table 2:** The variations of next of kin's perceptions of participation in the care of older persons in nursing homes

Category	A. Be present	B. Communicate	C. Monitor	D. Do practical tasks	E. Represent
Interview person					
1	x	x	x		
2	x	x	x	x	
3	x	x			
4	x	x			
5	x		x	x	x
6	x	x	x	x	
7	x	x	x		
8	x	x	x	x	x
9	x	x	x	x	
10	x	x	x	x	
11	x		x	x	
12	x	x	x	x	x
13	x	x	x		
14	x	x	x	x	x
15	x			x	
16	x	x		x	
17	x		x	x	
18	x	x		x	
Total	18	14	12	13	4

### The meaning of participation is to be present

Participation could mean *to be present* in different ways: physically, socially and mentally. The physical presence was described as going to the nursing home, which many of the next of kin did once a week, or calling the nursing home to talk with the older person. The presence in the older person's everyday life was perceived as very important at the end of life, particularly at the moment of death.

Yes, he's the only one I have left … and it feels important to me … to be present all the way to the end. (Interviewee 4, male 52 years.)

To be present mentally could be perceived as being socially present in the older person's life in different ways, for example having a conversation with the older person, looking at old pictures and/or reminiscing together. Another aspect was to include the older person in the life outside the nursing home by bringing the older person to their home, making sure that the older person was informed about events in other relatives’ lives or celebrations with the older person. Being present mentally and socially also included caring about the older person's well‐being in different ways.

### The meaning of participation is to communicate

Communicating with the staff, *to communicate*, was mentioned by the next of kin as another meaning of participation. The communication was perceived as both a two‐way communication and a one‐way communication. The next of kin provided examples of participation in planning meetings and engagement in a continuous dialogue with the nursing staff concerning the older person's care. Several next of kin reported the importance of cooperation and exchange of information between next of kin and nursing staff for their participation. Having daily conversations with the nursing staff was also mentioned.

Next of kin stressed the importance of obtaining information about the older person from the nursing home staff. Information about medical adjustments made by the doctor was perceived as particularly important. Next of kin also talked about the importance of receiving information about everyday happenings such as what the older person had been talking about, if the older person had said something special and what the older person's mood had been like. If anything remarkable had occurred, the next of kin were eager to be informed about events such as accidents or worsening of their general condition. Next of kin attributed a great deal of importance to acquiring information, with lack of or insufficient information being a barrier to participation.

… I think I have received a lot of information, and they are always there, they want to talk to you about what's happening. What they do with him [the older person], if I have not been there for a while … Then, I get like this whole feedback from them. (Interviewee 4, male 52 years.)

### The meaning of participation is to monitor

The meaning of participation for the next of kin could also be *to monitor* care in different ways so that the older person would not be neglected, or to improve the older person's well‐being. It could be to make sure that the older person receives more time with the contact person, and it could involve improving the cleanliness of the older person's apartment, changing the diet or spending more time outdoors.

The next of kin also perceived that the meaning of participation could be to monitor the older person's health and inform the staff at the nursing home what might be needed to improve it. Examples mentioned by next of kin were when they suspected that the older persons had pneumonia or that they wanted the older person to receive a percutaneous endoscopic gastrostomy because he or she had stopped eating.

So I interfere in care when I see something that makes my mother worse. (Interviewee 12, female 63 years.)

### The meaning of participation is to do practical tasks

In the category *to do practical tasks*, the meaning of participation had to do with anything from taking care of the older person's finances to assisting with personal care. The next of kin perceived that participation meant to be helpful in everyday tasks such as shopping, doing laundry, accompanying them on a doctor's visit and going with the older person to the hairdresser or dentist, but this participation could also involve feeding the older person, change incontinence pads and transferring the person from the bed to a chair. The next of kin often helped the older persons with practical tasks so the older person could live in a way that he or she was accustomed to or to experience some moments of enhanced well‐being, for example, being able to drink some alcohol or having their hair made to look nice. Other next of kin expressed a desire to pamper the older person as they reflected on everything this person had done for them and were determined to give something back. One next of kin expressed that the nursing staff appreciated these forms of practical help from the next of kin.

Then, of course, it's because, since Mom used to take care of herself, some things she wants to do or have in a special way. Some of those things we arrange…. like she wants fruit or she would like to drink wine… So, of course, we are the ones who take care of it because we are here. (Interviewee 10, female 68 years.)

### The meaning of participation is to represent the older person

*To represent* the older person was claimed by several next of kin as a meaning of participation. The next of kin believed that there often was a need to be an intermediator between the older person and the staff at the nursing home when the older person no longer could speak adequately for him‐ or herself. This intermediating role might be relevant if, for example, the food was too spicy, if the nursing staff did not come when the older person called for them, or if the older person did not want to bother the staff or cause trouble or discomfort.

When I have been here, I have been an intermediator [between the staff and the older person]. (Interviewee 14, female 70 years.)

### Hierarchy and relations between categories

**Figure 1:**
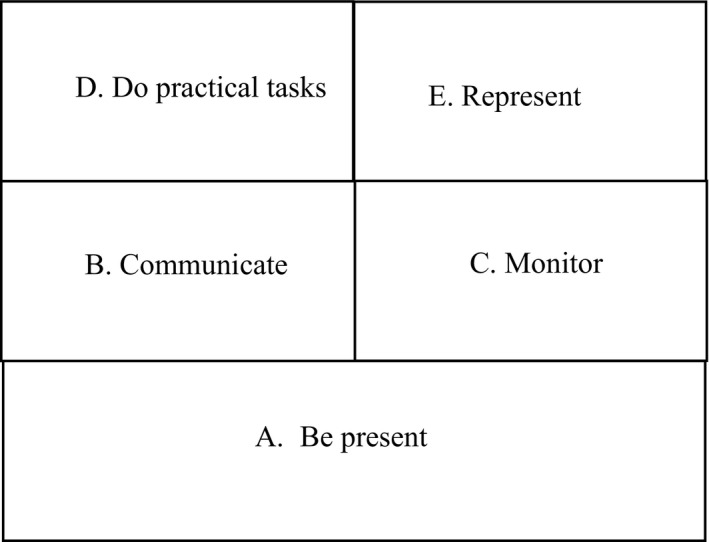
The hierarchy between the categories in the study.

A further step in the phenomenographic analysis is to present how the different categories in the outcome space are related to one another in a hierarchical way (see Fig. 1).

Category A, which signifies being present physically, mentally or socially, can be interpreted as a basic prerequisite for the next of kin's participation in the care of the older person at the nursing home. Category A thus becomes the foundation in the hierarchy of categories. To provide or obtain information about the older person's health or situation at the nursing home becomes the relational bond between categories B and C. Common to categories B and C is that different ways of receiving information about the older person are perceived as contributing to participation. However, the difference between categories B and C lies in the way in which the information is conveyed. In the case of category B, information about the older person derives from the nursing staff, while in the case of category C, such information is gathered by the next of kin without the influence of the staff, with the aim of making sure that the older person is not neglected and that his or her needs are fulfilled.

Efforts to improve the situation for the older person become the relational bond between categories D and E. In the case of category D, next of kin perceive that participation means helping with practical tasks such as shopping, doing laundry or having a meal, while in the case of category E, it is a question of the next of kin's assuming the role of an intermediary between the older person and the nursing staff to improve the care. Thus, a difference between the two categories is that the next of kin in category D take measures to improve the care themselves while those in category E aim to improve the care by informing the staff about what to do. In addition to the above associations between categories, it is possible to recognise that categories C and E both represent a lack of trust in the staff at the nursing home.

## Discussion

This study aimed to describe variations in next of kin's perceptions of the meaning of participation in the care of older persons living in nursing homes. We found multiple meanings of next of kin's participation in the care of the older person in nursing homes. The category *be present* can be interpreted as the basic prerequisite for the next of kin to participate further in the care of the older person. Being present encompassed a physical, social and mental presence in the old person's life. Our category *be present* is similar to Andershed and Ternestedt's 13 key concept *to be*, which describes next of kin's need for presence, to support and to accompany the older person in the final days of life and share each other's lifeworld. Andershed and Ternestedt's theory 13 describes three keywords of next of kin's involvement in care for a person at the end of life: to be, to know and to do. These categories were developed from interviews with six spouses of dying patients who were followed prospectively through different care cultures during 1–3 months after the patient's.

Death. Davies and Nolan 17 have shown that to be present is important since the next of kin has an intimate knowledge about the older person which can improve the older person's quality of life. The presence of the next of kin is also described as a calming influence on the old person 18.

Another aspect of Andershed and Ternestedt's 13 concept of *to be* is the communication between the older person and the next of kin. This is consistent with our study findings, which showed that to be present could mean to have a conversation with the older person, to look at old pictures and to remember old times together. One reason to be present can be that the next of kin is uncertain whether the older person receives the best possible care 5. Our study findings imply the relevance of nursing home staff's enabling the next of kin's presence and welcoming them at the nursing home.

Another perception of the meaning of participation was to *communicate*. Our category *communicate* resembles the concept of *to know* in Andershed and Ternestedt's theory 13 *. To know* included next of kin's getting information about the older person's situation. A previous study 12 showed that the next of kin believed that the best way to have influence in a nursing home was to be actively involved in different matters, but they also felt that they, rather than the nursing staff, had to initiate the communication because the older persons were afraid of disturbing the nursing staff. In another study 33, the next of kin experienced that if they were too passive, the nursing staff would only provide the most essential information. However, the study showed that the next of kin would like to have more spontaneous information about the older person's daily life, which is in line with our findings 33. Hence, to promote the participation of next of kin, nursing home staff should initiate communication and provide spontaneous information about the older person's daily life.Another way of collecting information which is connected to Andershed and Ternestedt's concept of involvement 13 is our category *monitor*. Monitoring, in contrast to *communication*, entails collecting information about the older person that does not involve the nursing staff. Monitoring has been described in previous studies 5, 16, 17, where next of kin have experienced that they need to supervise care to ensure that it is ‘correctly’ performed. However, maintaining control entails difficulties because next of kin are often afraid of being seen as supervising or upsetting the staff 17. On the other hand, next of kin also consider it as their right and responsibility to control what is going on at the nursing home 16.

The category *to act* is similar to Andershed and Ternestedt's 13 concept *to do,* which is task‐centred and means doing things for the person that he/she normally would do him‐/herself. The importance of performing tasks for the older person has also been emphasised in other studies 18, 19, 20, 22. If the next of kin does not have the ability to be a part of care, feelings of guilt and dissatisfaction can occur 5. Andershed and Ternestedt's 13 concept *to do* conveys that the next of kin can act as the old person's spokesperson, a situation which in our study was labelled *to represent*.

Our study reveals that participation in the care of older persons at nursing homes was perceived as having multiple meanings for the participants. Two ways of perceiving participation could be interpreted as being based on a lack of trust in the staff: to monitor and to be a mediator. Andershed and Ternestedt 13 emphasise that involvement ‘in the light’ is based on a trusting relationship with the staff, whereas involvement ‘in the dark’ is a result of insufficient collaboration. Other studies have shown that the relationship between the staff and the next of kin is of great importance with regard to the next of kin's feeling of being involved in the care 22, 34. These findings point to a need for the nursing staff to be approachable and to take time to talk to the next of kin. Research shows, however, that nurses do not consider the interaction with next of kin as a priority 35 and believe it is demanding because of a perceived lack of time. Nurses might stay away when a next of kin that they consider to be demanding visits 35, 36, and they lack training concerning interactions with next of kin 35. It is reasonable to believe that this viewpoint among the staff of next of kin might aggravate the next of kin's sense of belonging and created a mistrust in the staff. Thus, knowledge about participation of next of kin needs to be included in nursing training at all levels. A review of the literature 37 concerning interventions that promote the relationship between staff and next of kin suggests educating both staff and next of kin on relationship development, power and control issues, communication skills and negotiating techniques.

This study used a phenomenographic approach, which presupposes that a qualitative variation exists in how people experience phenomena 27. According to Sjöström and Dahlgren 38, phenomenography can be used to increase the understanding of how people understand the world. The results from phenomenographic studies can present different ways of understanding the world which can help healthcare professionals to meet different needs of patients and their next of kin. In accordance with the phenomenographic tradition, the participants in the study were strategically chosen with regard to age, gender and relation to the older person to achieve variation in perceptions. We did find considerable variations of perceptions in the data, which confirms that our strategic sampling was adequate. A limitation of the study is, however, that the next of kin included were chosen by a contact person at each nursing home based on our stipulated inclusion criteria. This may have affected who were asked and who were not asked by the contact person. Hence, the chosen participants might have been the ones who were felt most satisfied with care and/or those who visited most frequently. The fact that to be present was the most common perception of the meaning of participation in our study may be a reflection of the sample. However, it is reasonable that to be present is a common perception of the meaning of participation. Additionally, the contact persons who chose the participants were from different nursing homes in different cities and were aware of exclusion and inclusion criteria.

There are further limitations to the study that need to be addressed. The main question used in the interview, about next of kin's perceptions of the meaning of participation, was sometimes hard to understand by the participants. ‘Participation’ is an abstract concept, which made it difficult for some participants to express what participation meant to them. Many participants handled this by describing examples of how they participated in care.

The credibility of a study is often based on the descriptions of the analysis process which make it possible to replicate the study. Additionally, to strengthen the trustworthiness, the categories of the results need to be illustrated by quotations from the interviews. Through these, the reader is able to consider the relevance of the categories 38. Another aspect of the credibility is the extent to which another researcher would suggest the same categories after having worked with the same transcripts. In this study, the first and second authors (KE and SS) were not involved in the data collection, which reduced the risk of being influenced by the interview situations. Also, the two authors conducted the analysis separately before a joint discussion with the last author. Furthermore, three of the authors scrutinised the proposed categories before consensus was reached. Marton 39 argues, however, that the finding of the categories of description is a form of discovery, and discoveries do not have to be replicable. On the other hand, it must be possible to reach agreement concerning the categories if other researchers are to be able to use them.

## Conclusion

The study shows that there are multiple meanings of next of kin's participation in the care of older persons at nursing homes, with some participants having a more complex understanding of the phenomenon than others. Our results imply the relevance of nursing home staff's enabling next of kin's presence and welcoming them to be a part of care according to their own preferences. One way to enable them to be a part of care is to initiate communication. Nursing home staff's understanding of the meaning of the participation of next of kin in nursing homes could enable more person‐centred care and could be improved by integrating relevant knowledge into nursing training at all levels.

## Conflict of interest

The authors have no conflict of interest to declare.

## Author contributions

LB designed the study in collaboration with GA. LB and BW collected the data. KE and SS performed the qualitative analyses and wrote the first draft of the manuscript together with LB. The other authors (GA, PN, ÅA and BW) scrutinised the categories, before consensus was reached. Comments and improvement of the draft were made by GA and PN, ÅA and BW. All authors have approved the final version of the manuscript.

## Ethical approval

The study was designed in accordance with the ethical principles for medical research in the Declaration of Helsinki. Ethical permission for the study was obtained from the Regional Ethics Review Board in Lund, number 2015/69.

## Funding

This study was funded by The Swedish Research Council; The Vårdal Foundation; The Greta and Johan Kock Foundation; The Ragnhild and Einar Lundström foundation and The Ribbingska Memorial Foundation.
